# Oxygen Isotopic Fractionation of O_2_ Consumption by Methane and Ammonia Monooxygenases

**DOI:** 10.1021/acsenvironau.5c00180

**Published:** 2025-12-04

**Authors:** Carolina F. M. de Carvalho, Maartje A.H.J. van Kessel, Arjan Pol, Jakob Zopfi, Moritz F. Lehmann, Sarah G. Pati

**Affiliations:** † Department of Environmental Sciences, 27209 University of Basel, 4056 Basel, Switzerland; ‡ Department of Microbiology, 6029 Radboud University, 6525 AJ Nijmegen, The Netherlands; § Department of Environmental Geosciences, Centre for Microbiology and Environmental Systems Science, University of Vienna, 1090 Vienna, Austria

**Keywords:** oxygen isotope ratio, biogeochemical O_2_ cycling, methane oxidation, ammonia oxidation, isotopic fractionation

## Abstract

Understanding stable isotopic fractionation of dissolved O_2_ in aquatic environments is crucial to constrain and accurately model the processes responsible for biological O_2_ consumption, which are closely linked to the overall health of an ecosystem. This study aimed to investigate whether O_2_ consumption by microbial methane and ammonia oxidation may contribute to the observed discrepancy in O_2_ isotopic fractionation (^18^ϵ) between heterotrophic O_2_ respiration in laboratory incubations (−18 to −24 ‰) and *in situ* measurements of O_2_ consumption in lakes and oceans (−10 to −18 ‰). To estimate the *in vivo* ^18^ϵ values of soluble methane monooxygenase (sMMO), particulate methane monooxygenase (pMMO), and ammonia monooxygenase (AMO), which are the first enzymes required for the oxidation of methane and ammonia, experiments were performed with three methanotrophic bacteria and one comammox (complete-ammonia-oxidizing) bacterium. The resulting ^18^ϵ values for pMMO and AMO ranged from −18 ± 12 to −24 ± 5 ‰, not significantly different from ^18^ϵ values typical for heterotrophic respiration. The ^18^ϵ value determined for sMMO (−22 ± 2 ‰) was in the same range, yet more negative than the previously reported ^18^ϵ value for the isolated enzyme. Our results provide insights into the potential reaction mechanisms of pMMO and AMO and indicate that O_2_ consumption by sMMO, pMMO, or AMO cannot explain the observed discrepancy between *in situ* and laboratory ^18^ϵ values for “community” O_2_ consumption in aquatic environments. Instead, the apparent difference may be attributed to aspects involving substrate diffusion limitation.

## Graphical abstract

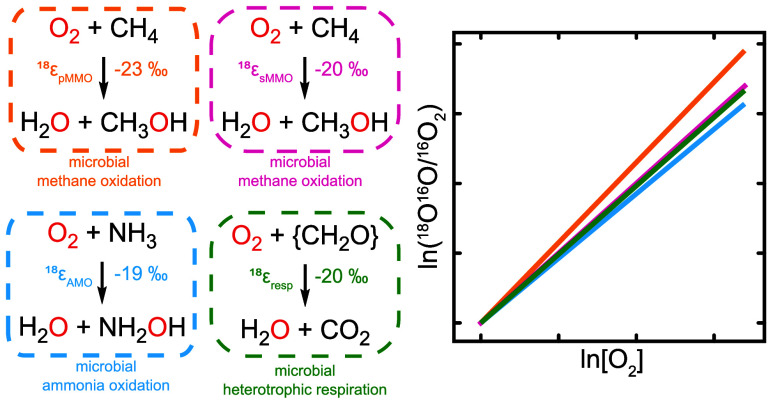

## 1 Introduction

Dissolved oxygen (O_2_) is the most important oxidizing agent in aquatic ecosystems, and its concentration controls the rate of many biotic and abiotic processes, from cellular metabolism to the biogeochemical cycling of elements. O_2_ concentrations and the redox state of an aquatic ecosystem represent important parameters that are directly linked to water quality and trophic status.^1^ As such, understanding O_2_ consumption and production dynamics in aquatic environments is imperative to comprehensively assess ecosystem functioning, health, and resilience. By analyzing the stable isotopic composition of dissolved O_2_ in aquatic environments, quantitative information can be gained on critical ecosystem processes producing or consuming O_2_, such as primary production and respiration.^2,3^ Changes in the isotopic composition of dissolved O_2_ (reported in the common δ^18^O notation, see eq 2) primarily result from the preferential reaction of ^16^O^16^O in biological and abiotic processes, leaving the remaining O_2_ enriched in ^16^O^18^O. Changes in δ^18^O values of O_2_, referred to as isotopic fractionation, can be quantified with ^18^ϵ values or apparent ^18^O kinetic isotope effects (^18^O-KIEs) according to eq 1,^4^

1 ϵ18=ln(δO18+1δO018+1)ln([O2]/[O2]0)=1O‐KIE18−1=1k16/k18−1

where δ^18^O and δ^18^O_0_ indicate the isotopic composition of O_2_ in a sample at a certain time point and in a reference sample representing initial conditions (*t*_0_), respectively. [O_2_]/[O_2_]_0_ is the fraction of the remaining O_2_ concentration upon partial consumption since *t*_0_, while ^16^ *k* and ^18^ *k* refer to the reaction rates of the light ^16^O^16^O and heavy ^18^O^16^O isotopologues, respectively.

Because ^18^ϵ values are typically characteristic for different processes causing changes in dissolved O_2_ concentrations, i.e., respiration, photosynthesis, and gas–water exchange, they can be used to identify, track, and quantify O_2_-consuming or producing processes in the environment. As such, several studies have applied stable isotope analysis of O_2_ to study O_2_ consumption dynamics in oceans and lakes.^5–10^ This approach usually involves determining ^18^ϵ values for respiration to estimate respiration rates, which then yield information about gross primary production. Typically, ^18^ϵ values are obtained from laboratory incubation experiments with water from the study site or by measuring the δ^18^O *in situ* as a function of depth and/or along other O_2_ concentration gradients. The “community” ^18^ϵ values obtained from incubation experiments have been shown to vary from −18 to −24 ‰,^5,10–13^ similar to the ^18^ϵ values determined for microbial respiration by the terminal cytochrome *c* oxidase (−18 to −21 ‰).^14–16^ In contrast, ^18^ϵ values determined from water column O_2_ isotope measurements tend to be considerably lower, ranging from −10 to −18 ‰.^6–8,10^ This may suggest that the enzyme-level O isotope effect associated with respiration is suppressed *in situ*, e.g., due to diffusion limitation. Alternatively, the observed lower water column ^18^ϵ values may also reflect the combined isotope effects of multiple distinct processes occurring in parallel within the same water mass. It is important to understand which processes modulate the observed water column ^18^ϵ value and thereby determine what causes the discrepancy between the ^18^ϵ values observed in incubation experiments versus the ^18^ϵ values obtained from δ^18^O and O_2_ concentration profiles in natural marine or lacustrine water columns. A better insight into this difference is essential for assessing O_2_-consuming processes and for accurately modeling and estimating O_2_ consumption dynamics in aquatic environments. So far, this discrepancy has been suggested to occur due to diffusion-limited O_2_ consumption in sediments,^7,17^ diffusion-limited respiration by microorganisms in sinking organic matter particles,^10,18^ mixing processes,^17^ or reduced biological O isotopic fractionation during O_2_ respiration at low temperatures^19^ as well as O_2_ consumption by biologically produced reactive oxygen species (ROS).^20^ Recently, we determined that the biological isotopic fractionation of O_2_ can range from −9 to −53 ‰, depending on the type of O_2_-consuming enzyme.^16^ Although aerobic microbial respiration typically dominates observed biological O_2_ consumption, other biological O_2_-consuming processes may also contribute significantly to the observed ^18^ϵ values, potentially explaining the observed ^18^ϵ value discrepancies if they impart different O isotope effects.

Besides aerobic microbial respiration, methane and ammonia oxidation are prevalent biological processes in aquatic environments, which may contribute significantly to the total “community” O_2_ consumption.^21–24^ For example, estimates of global methane oxidation in oceans and freshwaters translate to an annual O_2_ consumption of approximately 8·10^13^ g (assuming a 1O_2_:1CH_4_ stoichiometry).^25^ This value is 7 times larger than estimates of annual O_2_ consumption by photochemical reactions in marine waters, which is considered to be a major sink of O_2_ at least in surface waters.^26^ Aerobic methane- and ammonia-oxidizing microorganisms are not only widespread in the environment but also play dominant roles in specific environmental niches, where their metabolic activities significantly impact the biogeochemical cycling of carbon and nitrogen, respectively.^27–31^ Aerobic methane oxidation is the process by which methanotrophic bacteria oxidize methane (CH_4_) to carbon dioxide using O_2_ in a 2O_2_:1CH_4_ stoichiometry (see Figure 1).^32–34^ The first step of CH_4_ oxidation, i.e., to methanol (CH_3_OH), involves stoichiometric O_2_ consumption and is catalyzed by a methane monooxygenase (MMO) enzyme, either soluble methane monooxygenase (sMMO) or particulate methane monooxygenase (pMMO). sMMO is a cytosolic iron-dependent O_2_-consuming enzyme, whereas pMMO is membrane-bound, copper-dependent, and the most prevalent MMO among methanotrophic microorganisms.^35^ In aerobic ammonia oxidation, nitrifying microorganisms utilize O_2_ to oxidize ammonia (NH_3_) first to nitrite (NO_2_^–^) and subsequently to nitrate (NO_3_^–^).^36^ The overall stoichiometries of the consumption of O_2_ consumption vs NH_3_ oxidation are 1.5:1 and 2:1 for the oxidation to nitrite and for complete oxidation to nitrate, respectively (see Figure 1). The latter can involve two distinct organisms (ammonia-oxidizing bacteria or archaea in conjunction with nitrite-oxidizing bacteria) or a single comammox (complete-ammonia-oxidizing) bacterium.^37–39^ The first step of nitrification, ammonia oxidation to hydroxylamine (NH_2_OH), concomitant with O_2_ consumption, is performed by the membrane-bound, copper-dependent enzyme ammonia monooxygenase (AMO).^40,41^

**Figure 1:**
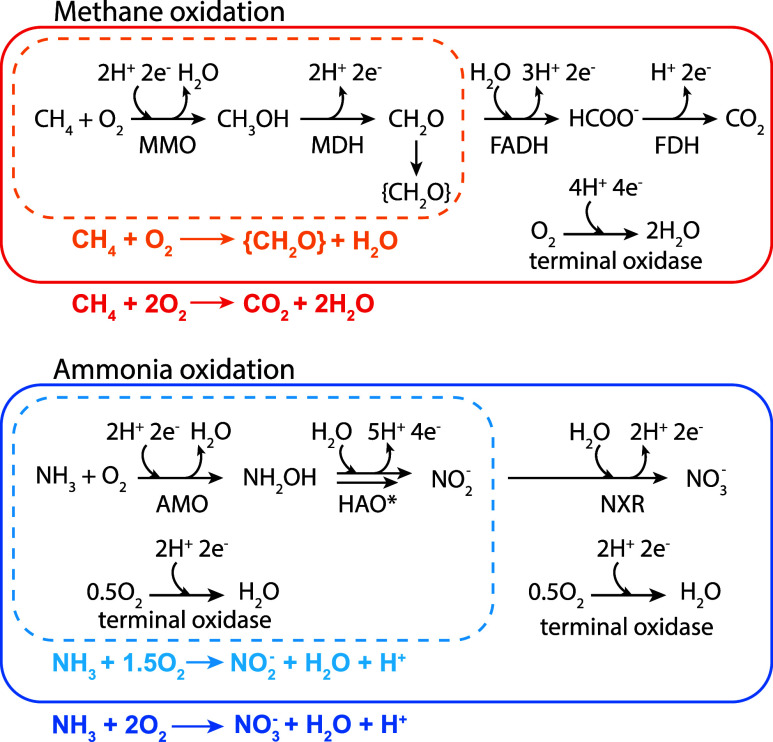
Key reaction steps in the oxidation of methane (CH_4_) by methanotrophic bacteria and ammonia (NH_3_) by ammonia-oxidizing microorganisms and comammox bacteria. Dashed-lined boxes highlight the reactions and overall stoichiometry of incomplete CH_4_ oxidation and cell biomass ({CH_3_O}) formation via methanol (CH_3_OH) and formaldehyde (CH_3_O), and NH_3_ oxidation to nitrite (NO_2_^–^) via hydroxylamine (NH_2_OH), respectively. Solid-lined boxes highlight the reactions and overall stoichiometries of complete CH_4_ oxidation to CO_2_ via formate (HCOO^–^) and NH_3_ oxidation to nitrate (NO_3_^–^), respectively. Enzymes involved in these reactions are methane monooxygenase (MMO), methanol dehydrogenase (MDH), formaldehyde dehydrogenase (FADH), formate dehydrogenase (FDH), ammonia monooxygenase (AMO), hydroxylamine oxidoreductase (HAO), nitrite oxidoreductase (NXR), and aerobic terminal oxidases. *We note that HAO only catalyzes the reaction of NH_2_OH to nitric oxide (NO), which is subsequently converted to NO_2_^–^.

Notably, pMMO and AMO share many characteristics, including membrane association, copper dependency, substrate specificity, putative subunit compositions, and a similar DNA sequence.^35,42,43^ Because they are membrane-bound, both enzymes have been proven difficult to purify while maintaining their activity,^35,39,44^ which has limited our understanding of their structure, mechanism, and the O isotopic fractionation they impart. In contrast, the cytosolic iron-dependent sMMO has been successfully purified with activity,^45^ and ^18^ϵ values between −15 and −17 ‰ have been determined for the isolated sMMO of *Methylococcus capsulatus*.^46^ Despite their prevalence and environmental relevance, ^18^ϵ values for pMMO or AMO have, to the best of our knowledge, not yet been determined. Other copper-dependent O_2_-consuming enzymes have been shown to express ^18^ϵ values between −9 and −22 ‰.^16^ An ^18^ϵ value closer to −9 ‰ for pMMO or AMO could explain the apparent under-expression of the respiration O isotope effect of approximately −20 ‰ in environments where aerobic methane and ammonia oxidation represent important processes. In contrast, an ^18^ϵ value closer to −22 ‰ for pMMO or AMO would make the O isotope effects for methane and ammonia oxidation indistinguishable from that of respiration, minimizing their potential to suppress the O isotope effect of water column respiration.

In this study, we determined the *in vivo* ^18^ϵ values for pMMO, sMMO, and AMO by performing experiments with the pMMO-containing *Methylomonas lenta* and *Methylotetracoccus oryzae*, sMMO-containing *Methylocella silvestris*, and with the AMO-containing *Nitrospira inopinata*, respectively. While *M. lenta* possesses the genes for both pMMO and sMMO,^47^ when grown in the presence of excess copper (typical culturing conditions), it expresses only pMMO.^35^ *M. oryzae* expresses only pMMO and *M. silvestris* only sMMO.^48,49^ By measuring the O_2_-to-CH_4_ and O_2_-to-NH_3_ consumption stoichiometries, we first determined the contribution of O_2_ consumption by pMMO, sMMO, and AMO, respectively, to the overall O_2_ consumption (i.e., relative to terminal respiratory oxidase activity). Second, based on the observed stoichiometries and measured ^18^ϵ values for total O_2_ consumption determined for each microorganism, we were able to calculate *in vivo* ^18^ϵ values for pMMO, sMMO, and AMO. Additionally, by supplying the methanotrophs with methanol as the substrate, we determined an isolated respiration value by the terminal cytochrome *c* oxidase. Our work provides novel insights into the previously unexplored O isotopic fractionation associated with the activity of two environmentally relevant O_2_-consuming enzymes, MMO and AMO. These new insights will be discussed in the context of whether O_2_ consumption by methane and/or ammonia oxidation in natural environments can explain the previously observed discrepancies in the “community” ^18^ϵ values for biological O_2_ consumption in the water column of lakes or oceans.

## 2 Materials and Methods

### 2.1 Bacterial Cultures

Pure cultures of *M. lenta* (LMG26260) and *M. oryzae* ^48^ were grown in 60 to 150 mL serum vials, containing 15 to 60 mL of dilute nitrate mineral salt solution (dNMS: 0.2 g L^–1^ MgSO_4_·7H_2_O, 0.006 g L^–1^ CaCl_2_·2H_2_O, 0.2 g L^–1^ NaNO_3_), supplemented with 5 mM phosphate buffer (pH 7.0), 0.1% (v/v) trace element solution I (10 g L^–1^ trisodium nitrilotriacetate and 5 g L^–1^ FeSO_4_·7H_2_O), and II (0.43 g L^–1^ ZnSO_4_·7H_2_O, 0.24 g L^–1^ CoCl_2_·6H_2_O, 0.99 g L^–1^ MnCl_2_·4H_2_O, 0.25 g L^–1^ CuSO_4_·5H_2_O, 0.22 g L^–1^ NaMoO_4_·2H_2_O, 0.19 g L^–1^ NiCl_2_·6H_2_O, 0.21 g L^–1^ NaSeO_4_·10H_2_O, 0.14 g L^–1^ H_3_BO_4_, and 0.24 g L^–1^ CeCl·6H_2_O), and a headspace of 20% CH_4_ in air.^50,51^ Cells were grown at 22 °C, with shaking at 150 rpm for 1 to 2 weeks. *M. silvestris* (DSMZ 15510) was cultured under similar conditions but at pH 5.8 and 26 °C. A pure culture of *N. inopinata* was grown aerobically in 1000 mL Schott bottles containing 200 mL of mineral medium (0.05 g L^–1^ KH_2_PO_4_, 0.075 g L^–1^ KCl, 0.05 g L^–1^ MgSO_4_·7H_2_O, 0.584 g L^–1^ NaCl) supplemented with 4 mM HEPES (pH 7.8) and 0.1% (v/v) of trace metal solutions (see composition above), at 37 °C, in the dark, without shaking.^52^ The pH was maintained by the addition of Na_2_CO_3_. All experiments were performed with actively growing batch cultures.

### 2.2 O_2_ Consumption Experiments with Methanotrophs

#### 2.2.1 Experiments with Methane as the Substrate

To determine the ^18^ϵ values of O_2_ consumption by different methanotrophs during CH_4_ oxiation, experiments were conducted in duplicate for all three methanotrophic species in a 60 mL incubation chamber that housed a membrane-inlet mass spectrometer (MIMS) probe following previously described procedures.^53,54^ The probe (3 mm in diameter with 4–16 perforated holes of 1 mm diameter each) was covered by silicon tubing and connected to the mass spectrometer, which was operated at a 40 μA emission current, via a 1/8- or 1/16-in. stainless-steel tube.

To monitor dissolved O_2_ concentrations the incubation chamber was equipped with an optical O_2_ sensor spot read by a FireStingO_2_ pro meter (PyroScience GmbH) with automated pressure, humidity, and temperature correction. A calibration point for 100% dissolved O_2_ was achieved by purging the water-filled chamber with air until stable readings (i.e., equilibrium concentrations) were obtained at the set experimental temperatures (268 and 260 μM at 25 and 28 °C, respectively; salinity 0 ppm).^55^ 0% dissolved O_2_ was assigned to stable low-level readings achieved after biological O_2_ consumption with excess substrate.

The chamber was placed on a magnetic stirring plate, and temperature was regulated by an external water bath connected to the chamber’s water jacket. Experiments with *M. lenta* and *M. oryzae* were conducted at 28 °C, experiments with *M. silvestris* at 25 °C. Liquid and gas could be introduced into the chamber via a central hole in the piston (0.8 mm diameter), using metal capillaries or gastight syringes. Any gas headspace was eliminated before the start of the measurement. The central piston hole was sealed with a 1/16-in. PEEK tube (0.76 mm inner diameter) Luer-locked to a stainless-steel needle (0.41 mm o.d.), through which samples were obtained by lowering the piston. When taking a sample, the first mL of solution was always discarded, then the PEEK tube-attached needle was injected into a 12 mL Exetainer (Labco Limited), and 3 to 7 mL of the sample was transferred by lowering the piston. Prior to starting an experiment, Exetainers were closed with chlorobutyl septa and screw caps, purged with He gas for 1 h, and amended with 200 μL of 3.2 M HCl to inactivate the bacteria upon sample transfer. To ensure equal headspace pressure in the Exetainers with different sample volumes, Exetainer septa were pierced with a stainless-steel needle (0.45 mm o.d.) connected with a T-piece to a slow He flow, and an open outlet submerged under 10 cm of water during sample injection, as described in de Carvalho et al.^16^ Procedural blanks were prepared by filling the incubation chamber with N_2_-purged (<0.1% O_2_) water and transferring 3 to 7 mL of this O_2_-free water to He-purged Exetainers containing HCl, as described above. After injection, Exetainers containing samples or procedural blanks were mixed gently and stored upside down in the dark, with the lid submerged under water, until isotope analysis was performed within 16 days after sample/blank collection (see Section 2.4). We experimentally verified that within this time frame isotopic fractionation of O_2_ consumption can be determined reliably.

CH_4_ concentrations were determined from the MIMS mass-to-charge (*m*/*z*) signal at *m*/*z* 15 (i.e., CH_3_^+^, a fragment formed in the ion source). *m*/*z* 15 was selected over *m*/*z* 16 due to lower background signals. A calibration was performed by sequentially injecting two 1 mL aliquots of CH_4_-saturated water (1.5 mM CH_4_)^56^ into the water-filled, initially CH_4_-free chamber while continuously recording a signal. This procedure resulted in three calibration points at 0, 30, and 60 μM dissolved CH_4_. The CH_4_-saturated water was prepared at room temperature in a sealed bottle under a known pressure (1.10–1.14 bar) of pure CH_4_. Accuracy at higher CH_4_ concentrations was verified by equilibrating the liquid filled chamber with 30% CH_4_ in air, resulting in a dissolved CH_4_ concentration of 450 μM.

For each experiment, the chamber was filled with 52–57 mL of dNMS medium. The medium was purged with air to obtain air-saturated O_2_ concentrations. CH_4_ gas was introduced into a 1 to 2 mL headspace to achieve an initial dissolved CH_4_ concentration between 250 and 450 μM. The generation of a CH_4_ headspace led to a decrease of the dissolved O_2_ concentrations to 184 to 214 μM. Once the desired experimental starting conditions were achieved, the CH_4_ headspace was removed, and 0.1 to 1 mL of a bacterial suspension (obtained by centrifugation of 30 to 120 mL of active culture at 4000 rpm and subsequent resuspension in fresh medium) was added to start the reaction. Experiments were stirred at 500 rpm, except when adding bacterial suspensions. The first sample (3 mL) was taken immediately after adding bacterial suspension at a dissolved O_2_ concentration of 193 ± 8 μM. Four additional samples (3–7 mL) were taken at decreasing O_2_ concentrations down to 53 ± 2 μM.

#### 2.2.2 Experiments with Methanol as the Substrate

To determine ^18^ϵ values representative for O_2_ consumption by methanotrophic respiration alone (i.e., excluding O_2_ consumption by pMMO or sMMO), methanol was used as the substrate. Additionally, to assess the potential impact of background O_2_ consumption by the MIMS probe (up to 5%) on the measured δ^18^O values, replicate experiments were conducted in two distinct setups: (1) in the 60 mL incubation chamber with an active MIMS probe, and (2) in a 50 mL gastight glass syringe, where background O_2_ consumption by the MIMS probe was absent.

Experiments were conducted in both the incubation chamber with the MIMS probe, as described above, but with 2.3 mM methanol instead of CH_4_, and in the gastight glass syringe, containing a stir bar and optical sensor spots for measuring O_2_ concentrations and temperature (PyroScience GmbH), at room temperature as described in de Carvalho et al.^16^ In brief, the syringe was filled with 50 mL of air-saturated dNMS medium containing 0.1 to 1 mL of concentrated bacterial biomass. A 100 μL gastight glass syringe was used to add an aqueous solution of methanol (for a final concentration of 2.3 mM) through the Luer-tip opening of the syringe to initiate the reaction. Immediately after methanol addition, a stainless-steel needle (0.8 mm o.d.) was attached to the Luer-Lock tip of the syringe and pushed into a 12 mm thick chlorobutyl stopper to limit O_2_ exchange with the atmosphere. Six samples of 3–7 mL were taken from this syringe reactor and transferred to He-purged Exetainers as described in Section 2.2.1. Residual O_2_ concentration in the samples typically ranged from 200 to 50 μM. A control sample was prepared by transferring 3 mL of remaining assay solution without methanol with a 10 mL gastight glass syringe into a He-purged Exetainer containing 200 μL of 3.2 M HCl. These control samples were assumed to be representative of the initial O_2_ concentration (270 ± 5 μM) and isotopic composition. Blanks were prepared by transferring 3 to 7 mL of N_2_-purged water with a 50 mL gastight glass syringe from closed serum bottles into He-purged Exetainers. Exetainers containing samples and blanks were stored as described above.

### 2.3 O_2_ Consumption Experiments with *N. inopinata*

To determine the ^18^ϵ values of O_2_ consumption by the comammox bacterium *N. inopinata* during NH_3_ oxidation, experiments were conducted in a 50 mL gastight glass syringe, as described in Section 2.2.2. The assay solution was prepared by adding 15 mL of ammonium (NH_4_^+^)-free mineral medium supplemented with 20 mM HEPES (pH 7.8) to 45 mL of an active *N. inopinata* culture. The syringe was filled headspace-free and placed onto a stirring plate (500 rpm) inside an incubator (37 °C). The assay solution contained approximately 100 μM NH_4_^+^, but an additional 500 μM NH_4_^+^ was introduced through the Luer-lock tip of the syringe at the start of the reaction. Six 2 mL samples were taken for NH_3_, NO_3_^–^, and NO_2_^–^ concentration measurements, and six additional samples (3–7 mL) were taken for O_2_ isotope analysis. Samples for NH_3_, NO_3_^–^, and NO_2_^–^ concentration measurements, collected in 2 mL Eppendorf tubes, were always taken directly before the samples for isotope analysis. The latter were collected into 12 mL He-purged Exetainers as described in Section 2.2.1, containing 50 μL saturated HgCl_2_ solution. Adding HCl did not completely stop the reaction; instead, it led to additional O_2_ consumption likely due to the reaction with nitrous acid. All handling of HgCl_2_ was performed with appropriate caution, and all solutions were disposed of as hazardous waste. The first samples were taken immediately (i.e., approximately 30 s) after the NH_4_^+^ addition, representing the initial O_2_, (NH_4_^+^ + NH_3_), NO_3_^–^, and NO_2_^–^ concentrations and δ^18^O values of O_2_. The remaining five samples were collected at O_2_ concentrations of approximately 150, 120, 90, 70, and 50 μM. Samples collected for the dissolved inorganic nitrogen species were immediately passed through a sterile membrane filter (Sarsted, Filtropur, 0.2 μM pore size). Concentrations of (NH_4_^+^ + NH_3_) were determined by Hach Lange ammonium tests (0.2 to 2 mg L^–1^ NH_4_^+^ + NH_3_, HACH). NO_2_^–^ was determined colorimetrically using sulfanilamide and *N*-(1-Naphthyl)­ethylenediamine according to Hansen and Koroleff.^57^ NO_*x*_ (i.e., NO_2_^–^ + NO_3_^–^) was determined using an NO_*x*_ TELEDYNE T200 Analyzer (TELEDYNE API, CA, USA) involving the reduction of NO_*x*_ with hot acidic Vanadium­(III) solution to NO gas and subsequent chemiluminescence detection.^58^

### 2.4 Stable Isotope Analysis

The δ^18^O values of O_2_ were measured in the headspace of the 12 mL Exetainers using a GasBench II coupled via a Conflo IV to a Delta V Plus isotope ratio mass spectrometer (Thermo Fisher Scientific) as described by de Carvalho et al.^59^ and reported in permil (‰ ± one standard deviation) relative to the international measurement standard Vienna Standard Mean Ocean Water (VSMOW), according to eq 2.

2 δO18=(O18/O16)sample(O18/O16)VSMOW−1

where (^18^O/^16^O)_sample_ and (^18^O/^16^O)_VSMOW_ are the ratios of heavy to light isotopes in O_2_ in a sample and in VSMOW, respectively. In brief, seven 100 μL injections were made from each Exetainer headspace onto a 60 m Rt-Molsieve 5 Å PLOT column (Restek from BGB Analytik, 0.32 mm ID, 30 μm film thickness) maintained at 25 °C. Each GC/IRMS measurement sequence comprised 7–16 experimental samples, 10–15 procedural blanks, five water standards, and three air standards. Air standards were evenly distributed across the sequence and consisted of 150 μL of ambient air in 12 mL of He. Air standards were used to verify the absence of any instrument drift and to perform a one-point calibration of the δ^18^O values on the VSMOW scale. In recent work, we could demonstrate that the error introduced when using a one-point calibration with air is negligible for δ^18^O values of O_2_.^59^ The δ^18^O value of O_2_ in air was assumed to be 23.8 ‰.^60–62^ Procedural blanks were used to correct the measured δ^18^O values for blank contributions.^63^ Water standards (Exetainers containing different amounts of air-saturated water) were used to correct δ^18^O values for instrument linearity (change in δ values with signal size).^64^

### 2.5 Data Analysis

^18^ϵ values were determined from the slope obtained through the linear regression fit according to eq 1 and are expressed in permil (‰). For experiments conducted in the 60 mL incubation chamber (Section 2.2.1), the consumption rates of O_2_ and CH_4_ were corrected for background consumption by the MIMS probe. The O_2_-to-CH_4_ and O_2_-to-NH_3_ consumption stoichiometries were determined based on the measured concentrations of O_2_ versus CH_4_ or NH_3_, respectively. All linear regressions were performed with R version 4.3.1 ^65^ and the lm and confint functions. Unless noted otherwise, errors are reported as 95% confidence intervals.

## 3 Results and Discussion

### 3.1 Isotopic Fractionation Associated with O_2_ Consumption by Methanotrophs

During O_2_ consumption experiments with methanotrophs when using CH_4_ as the substrate, the δ^18^O values of the unreacted dissolved O_2_ increased with progressing O_2_ consumption, consistent with the Rayleigh-type dynamics predicted by eq 1. As an example, Figure 2 shows data from one experiment with *M. lenta*, including the measured O_2_ and CH_4_ concentrations (Figure 2A) and the changes in δ^18^O of the remaining O_2_ over time (Figure 2C). An O_2_:CH_4_ consumption stoichiometry of 1.49 ± 0.01 was determined based on the slope of the linear regression between O_2_ and CH_4_ concentrations (see Figure 2B). The fact that this stoichiometry is between 1 and 2 indicates that both complete CH_4_ oxidation to CO_2_ (for energy generation) and incomplete CH_4_ oxidation in combination with cell biomass formation (see Figure 1) occurred during this experiment. In addition, Figure 2D shows the linearized isotope and concentration data, from which an ^18^ϵ_bulk_ value of −23 ± 3 ‰ was calculated. ^18^ϵ_bulk_ values, which represent bulk O isotopic fractionation of total O_2_ consumption, were between −20 ± 1 ‰ and −23 ± 3 ‰ for all experiments with methanotrophs using CH_4_ as the substrate (see upper-row panels in Figure 3 and Table 1). ^18^ϵ_resp_ values determined in experiments with methanotrophs using methanol as the substrate ranged between −19.0 ± 0.7 ‰ and −22 ± 3 ‰ (see bottom-row panels in Figure 3 and Table 1), in agreement with previous studies on terminal respiration in microbial cultures and pure enzymes.^5,10,13,14,16^ The ^18^ϵ_resp_ value determined for *M. lenta* in the presence of the MIMS probe (−22 ± 3 ‰, *n* = 1; Figure 3, red circles) was identical within error to the ^18^ϵ_resp_ value determined in the syringe reactor (−20 ± 1 ‰, *n* = 2; Figure 3, orange and green circles), confirming that O_2_ consumption by the MIMS probe (up 5% of the observed O_2_ consumption rate) did not significantly affect the measured isotopic fractionation of O_2_. For further considerations, average ^18^ϵ_resp_ values per species were used (see Table 1).

**Figure 2:**
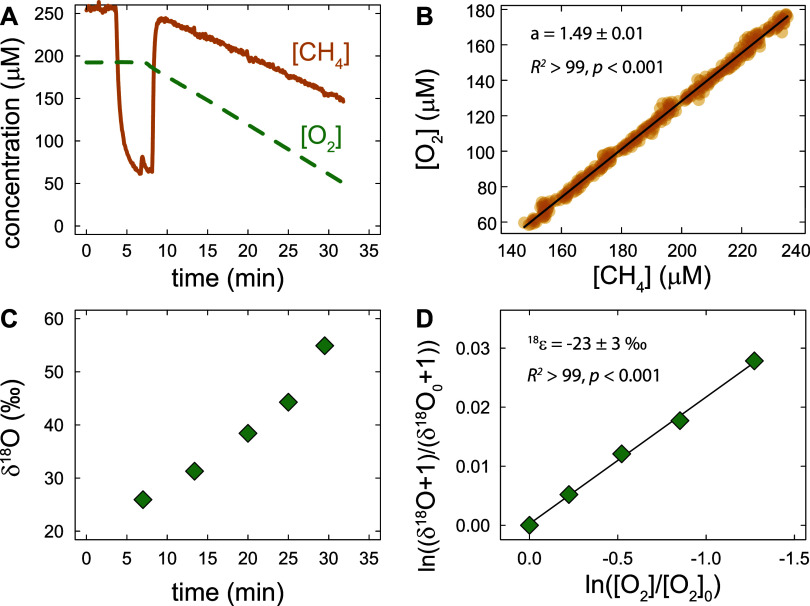
O_2_ and CH_4_ consumption and isotopic fractionation of O_2_ during a single experiment with *M. lenta*. (A) Change in concentrations of dissolved O_2_ (dashed line) and CH_4_ (solid line) over time. The dip in CH_4_ concentration between 5 and 10 min was due to a measurement artifact when stirring was paused to add bacterial biomass to start the reaction. (B) O_2_ versus CH_4_ concentrations from panel A. The black line indicates a linear regression fit with a slope of 1.49 ± 0.01 representing the O_2_:CH_4_ consumption stoichiometry. (C) δ^18^O values of unreacted O_2_ measured in discrete samples over time. (D) Linearized and normalized data (δ^18^O vs [O_2_]) from panels A and C, where [O_2_]_0_ and δ^18^O_0_ represent the concentration and δ^18^O value, respectively, of O_2_ at the beginning of the experiment. The solid line shows a linear regression fit according to eq 1, from which an ^18^ϵ value of −23 ± 3 ‰ was obtained.

**Figure 3:**
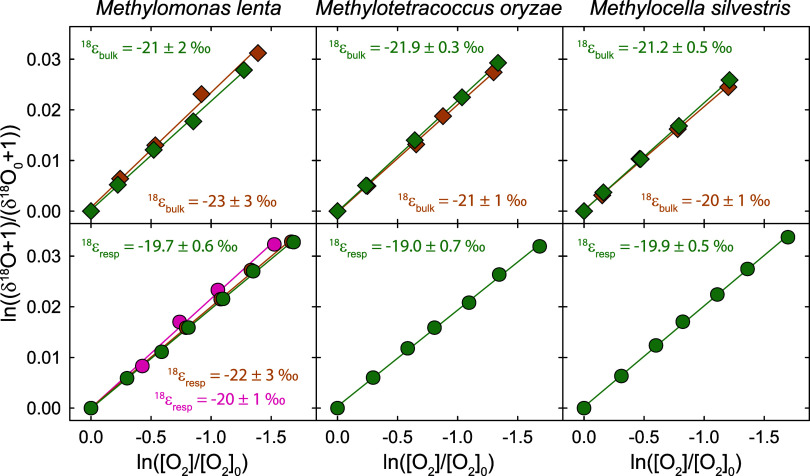
Log-normalized change in δ^18^O values vs log-normalized concentrations of O_2_ ([O_2_]) from experiments with different methanotrophs, where [O_2_]_0_ and δ^18^O_0_ are the concentration and δ^18^O value of O_2_ at the beginning of an experiment. The upper-row panels show results from experiments performed with CH_4_ as the substrate. The bottom-row panels show results from experiments performed with methanol as the substrate. Replicate experiments are colored green, orange, and red. Experiments with CH_4_ and one replicate experiment with methanol and *M. lenta* (red circles) were performed in the incubation chamber with the MIMs probe. All other experiments with methanol were performed in a syringe reactor (see Section 2.2 for details). The slopes of the linear regressions are reported for each experiment in ‰ as ^18^ϵ_bulk_ (upper row) and ^18^ϵ_resp_ (lower row). All linear regressions were significant (*p* < 0.001) and had adjusted *R* ^2^ values ≥ 0.99.

**Table 1:** O_2_:CH_4_ Consumption Stoichiometries (Δ­[O_2_]/Δ­[CH_4_]), the Fraction of O_2_ Consumed by MMO (*f*_MMO_), ^18^ *ϵ* Values Determined with CH_4_ (^18^ *ϵ*_bulk_) and Methanol (^18^ *ϵ*_resp_) as the Substrate, and the Calculated O Isotope Effects Imparted by Methane Monooxygenase (as ^18^ *ϵ*_MMO_ and Average Apparent ^18^O-KIE, see eq 1) for Three Methanotrophic Bacteria

species	Δ[O_2_]/Δ[CH_4_] (−)	*f*_MMO_ (−)	^18^ϵ_bulk_ (‰)	^18^ϵ_resp_ (‰)	^18^ϵ_MMO_ (‰)	^18^O-KIE (−)
*M. lenta*	1.358 ± 0.009	0.736 ± 0.005	–21 ± 2	–20.1 ± 0.9	–22 ± 3	1.023 ± 0.004
	1.49 ± 0.01	0.670 ± 0.005	–23 ± 3		–24 ± 5	
*M. oryzae*	1.266 ± 0.007	0.790 ± 0.004	–21.9 ± 0.3	–19.0 ± 0.7	–23 ± 1	1.023 ± 0.002
	1.323 ± 0.009	0.756 ± 0.005	–21 ± 1		–22 ± 2	
*M. silvestris*	1.49 ± 0.02	0.673 ± 0.007	–21.2 ± 0.5	–19.9 ± 0.5	–22 ± 3	1.022 ± 0.002
	1.341 ± 0.008	0.746 ± 0.004	–20 ± 1		–21 ± 1	

As stated above, ^18^ϵ_bulk_ values represent the bulk O isotopic fractionation of total O_2_ consumption, which integrates the O isotopic fractionation associated with both MMO activity (^18^ϵ_MMO_) and terminal respiration (^18^ϵ_resp_). In *M. lenta* and *M. oryzae*, MMO activity is ascribed to pMMO, while in *M. silvestris*, it is ascribed to sMMO. Using the observed O_2_:CH_4_ consumption stoichiometry, we estimated the relative contribution of the two O_2_ consuming processes (i.e., the fractional contribution of MMO, *f*_MMO_, versus respiration, *f*_resp_) to total O_2_ consumption, which was then used to calculate ^18^ϵ_MMO_ from ^18^ϵ_bulk_ and ^18^ϵ_resp_ as outlined in eq 3.

3 ϵbulk18=fMMO·ϵMMO18+fresp·ϵresp18=fMMO·ϵMMO18+(1−fMMO)·ϵresp18

We considered the plausible stoichiometries for CH_4_ oxidation to range from 1CH_4_:2O_2_, during complete oxidation of CH_4_ to CO_2_, to 1CH_4_:1O_2_, for incomplete oxidation of CH_4_ and cell biomass formation (see Figure 1). The O_2_:CH_4_ consumption stoichiometries, determined in this study, ranged from 1.266 ± 0.007 to 1.49 ± 0.02 (see Table 1), indicating a mix of complete and incomplete CH_4_ oxidation, consistent with previous studies.^66,67^ We can use these stoichiometries as a direct proxy for the fraction of O_2_ consumption by MMO (see eq 4) because based on the reactions shown in Figure 1, the amount of CH_4_ consumed (Δ­[CH_4_]) is equal to the amount of O_2_ consumed by MMO (Δ­[O_2_]_MMO_) and the amount of total O_2_ consumed (Δ­[O_2_]) is equal to the sum of O_2_ consumed by respiration (Δ­[O_2_]_resp_) and MMO.

4 $$\frac{\Delta \left[O_{2}\right]}{\Delta \left[{\mathrm{CH}}_{4}\right]}=\frac{\Delta {\left[O_{2}\right]}_{\mathrm{MMO}}+\Delta {\left[O_{2}\right]}_{\mathrm{resp}}}{\Delta {\left[O_{2}\right]}_{\mathrm{MMO}}}=\frac{1}{f_{\mathrm{MMO}}}$$

We can thus calculate O isotopic fractionation associated with O_2_ consumption by MMO for the three methanotrophs with eqs 3 and 4 and the measured parameters O_2_:CH_4_ stoichiometry, ^18^ϵ_bulk_ and ^18^ϵ_resp_. The resulting average ^18^ϵ_MMO_ values were −22 ± 2 ‰ for *M. silvestris*, −23 ± 2 ‰ for *M. oryzae*, and −23 ± 4 ‰ for *M. lenta* (see Table 1). While not significantly different, there is a slight difference in isotopic fractionation between pMMO (^18^ϵ_MMO_ = −23 ± 3 ‰) and sMMO (^18^ϵ_MMO_ = −22 ± 2 ‰).

### 3.2 Isotopic Fractionation Associated with O_2_ Consumption by *N. inopinata*

^18^ϵ_bulk_ values associated with O_2_ consumption by the complete-ammonia oxidizer *N. inopinata* were determined in experiments analogously to those with methanotrophs, except that NH_3_ consumption was determined from NH_4_^+^ concentrations in discrete samples at different time points. The O_2_:NH_3_ consumption stoichiometries were 1.7 ± 0.5 and 1.5 ± 0.7, respectively, and the corresponding ^18^ϵ_bulk_ values were −19 ± 2 ‰ and −19.1 ± 0.7 ‰ (see Figure 4 and Table 2), similar to the ^18^ϵ_bulk_ for methanotrophic O_2_ consumption. The fact that average NO_2_^–^ (62 ± 54 μM) and NO_*x*_ production (56 ± 103 μM) were both comparable to average NH_3_ consumption (96 ± 43 μM) together with an average O_2_:NH_3_ stoichiometry of 1.6 suggests that the majority of NH_3_ was oxidized only to NO_2_^–^ (partial nitrification) during the course of the experiments (see Figure 1). Note that the poor precision of the N-species concentration measurements are due to the fact that initial concentrations of NH_4_^+^, NO_2_^–^, and NO_3_^–^ (approximately 0.6, 1.3, and 3.9 mM, respectively) were high relative to the changes measured in these concentrations.

**Figure 4:**
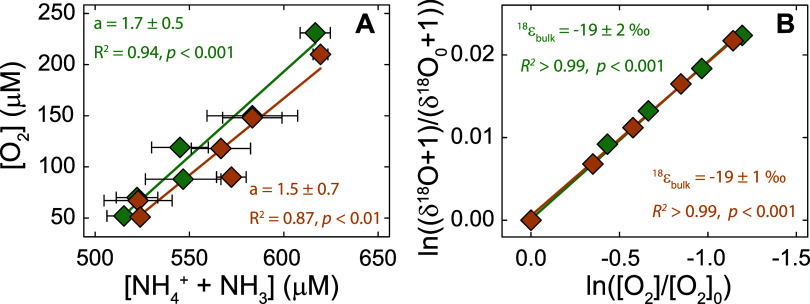
(A) O_2_ vs (NH_4_^+^ + NH_3_) concentrations in two replicate experiments with *N. inopinata*. The NH_4_^+^ concentrations were determined from duplicate measurements at different time points of the reaction (error bars represent standard deviations). The solid lines show a linear regression fit of the data in each experiment, with the slopes representing the respective O_2_:NH_3_ consumption stoichiometries. (B) Log-normalized change in δ^18^O values vs log-normalized concentration of O_2_ for experiments with *N. inopinata*. The solid lines show a linear regression fit according to eq 1, with slopes indicating ^18^ϵ_bulk_ values.

**Table 2:** O_2_:NH_3_ Consumption Stoichiometries (Δ­[O_2_]/Δ­[NH_3_]), the Fraction of O_2_ Consumed by AMO (*f*_AMO_), ^18^ *ϵ* Values Determined *In Vivo* (^18^ *ϵ*_bulk_), and the Calculated O Isotope Effects Imparted by Ammonia Monooxygenase (as ^18^ *ϵ*_AMO_ and Average Apparent ^18^O-KIE, see eq 1) for *N. inopinata*

species	Δ[O_2_]/Δ[NH_3_] (−)	*f*_AMO_ (−)	^18^ϵ_bulk_ (‰)	^18^ϵ_AMO_ (‰)	^18^O-KIE (−)
*N. inopinata*	1.5 ± 0.7	0.7 ± 0.3	–19.1 ± 0.7	–19 ± 14	1.019 ± 0.014
	1.7 ± 0.5	0.6 ± 0.2	–19 ± 2	–18 ± 12	

Similar to O_2_ consumption by methanotrophs, the average ^18^ϵ_bulk_ value for O_2_ consumption by *N. inopinata* reflects the combined O isotopic fractionation imparted by both AMO and respiration. As with methanotrophs, where experiments with methanol were used to isolate the respiratory ^18^ϵ_resp_ value by excluding MMO-driven O_2_ consumption, a similar approach could theoretically be applied to *N. inopinata* by using hydroxylamine as an intermediate substrate to bypass AMO activity. However, due to the reactivity of hydroxylamine, an isolated respiration ^18^ϵ_resp_ value for *N. inopinata* could not be determined. Nevertheless, ^18^ϵ_resp_ for *N. inopinata* can be reasonably approximated based on known properties of its respiratory machinery. Notably, *N. inopinata* lacks the canonical terminal copper-heme-dependent cytochrome *c* oxidase, and instead expresses a heme-dependent cytochrome *bd*-like oxidase.^68,69^ Stolper et al.^19^ demonstrated that the ^18^ϵ_resp_ values obtained for *Escherichia coli* mutant strains containing either only cytochrome *c* oxidase, only cytochrome *bd*-I oxidase, or only cytochrome *bd*-II oxidase, were indistinguishable within error, ranging from −14.9 to −15.5 ‰. Although these ^18^ϵ_resp_ values are lower than those typically reported for isolated cytochrome *c* oxidase, or microbial respiration more broadly,^14,15,70,71^ they nevertheless suggest that the cytochrome *bd*-like oxidase in *N. inopinata* may impart ^18^ϵ_resp_ values similar to those of more common respiratory terminal oxidases found in most bacteria (i.e., approximately −20 ‰).

Similar to the approach used for the methanotrophs in this study, the O_2_:NH_3_ consumption stoichiometry was employed to calculate the relative contributions of AMO activity versus respiration to total O_2_ consumption and, in turn, to calculate ^18^ϵ_AMO_ from the observed ^18^ϵ_bulk_ values and an assumed ^18^ϵ_resp_ of −20 ‰ with eq 5.

5 ϵbulk18=fAMO·ϵAMO18+fresp·ϵresp18=Δ[NH3]Δ[O2]·ϵAMO18+(1−Δ[NH3]Δ[O2])·ϵresp18

We note that the calculated average ^18^ϵ_AMO_ value of −19 ± 13 ‰ (Table 2) is contingent on the assumed ^18^ϵ_resp_, which was not independently determined (unlike in our methanotroph experiments) but instead derived from the existing literature. Nevertheless, varying the assumed ^18^ϵ_resp_ value by ±5 ‰ still yields ^18^ϵ_AMO_ estimates (−15 to −22 ‰) that fall within the typical range reported for terminal aerobic respiration (^18^ϵ_resp_). The high uncertainty of the ^18^ϵ_AMO_ estimate is due to the elevated 95% confidence intervals of the O_2_:NH_3_ stoichiometries (Table 2), and thus it is a consequence of the errors associated with NH_3_ concentration measurements.

### 3.3 Kinetic Isotope Effects and O_2_ Activation Mechanisms of Enzymes Involved in Methane and Ammonia Oxidation

The *in vivo* ^18^ϵ_MMO_, ^18^ϵ_AMO_, and ^18^ϵ_resp_ values determined in this study can be converted into average apparent ^18^O-KIEs of 1.023 ± 0.003, 1.022 ± 0.002, 1.019 ± 0.014, and 1.020 ± 0.001 (Tables 1 and 2) for the reduction of O_2_ by pMMO, sMMO, AMO, and terminal oxidase, respectively. ^18^O-KIEs can be used as mechanistic probes to assess the rate-limiting steps in O_2_-consuming enzymatic reactions and thus shed light on the catalytic cycle of such enzymes.^72^ This is of particular interest for pMMO and AMO, whose catalytic cycles have not been well studied. Because pMMO, sMMO, AMO, and terminal oxidase represent three different classes of O_2_-consuming enzymes with distinct active-site structures, it is surprising to see their respective ^18^O-KIEs fall within error of each other. To better link these values with the potentially rate-limiting reaction steps, a closer look at these enzyme’s catalytic cycles is required.

Cytochrome *c* oxidase, the most prevalent terminal oxidase, is a well-studied heme-copper-dependent enzyme, in which the heme a3 subunit initially binds O_2_ reversibly, forming an iron-superoxo intermediate (see Figure 5a).^73^ In the subsequent step, which is suggested to be rate-limiting, a hydrogen atom abstraction from an adjacent tyrosine residue by this iron-superoxo species, concomitant with O–O bond cleavage, leads to the formation of an iron-oxo and a copper-hydroxo species.^73^ The measured ^18^O-KIE of 1.020 ± 0.001 for terminal oxidases is in agreement with such a rate-limiting hydrogen atom abstraction step by a metal-superoxo intermediate as well as with a rate-limiting iron-oxo species formation.^74,75^

**Figure 5:**
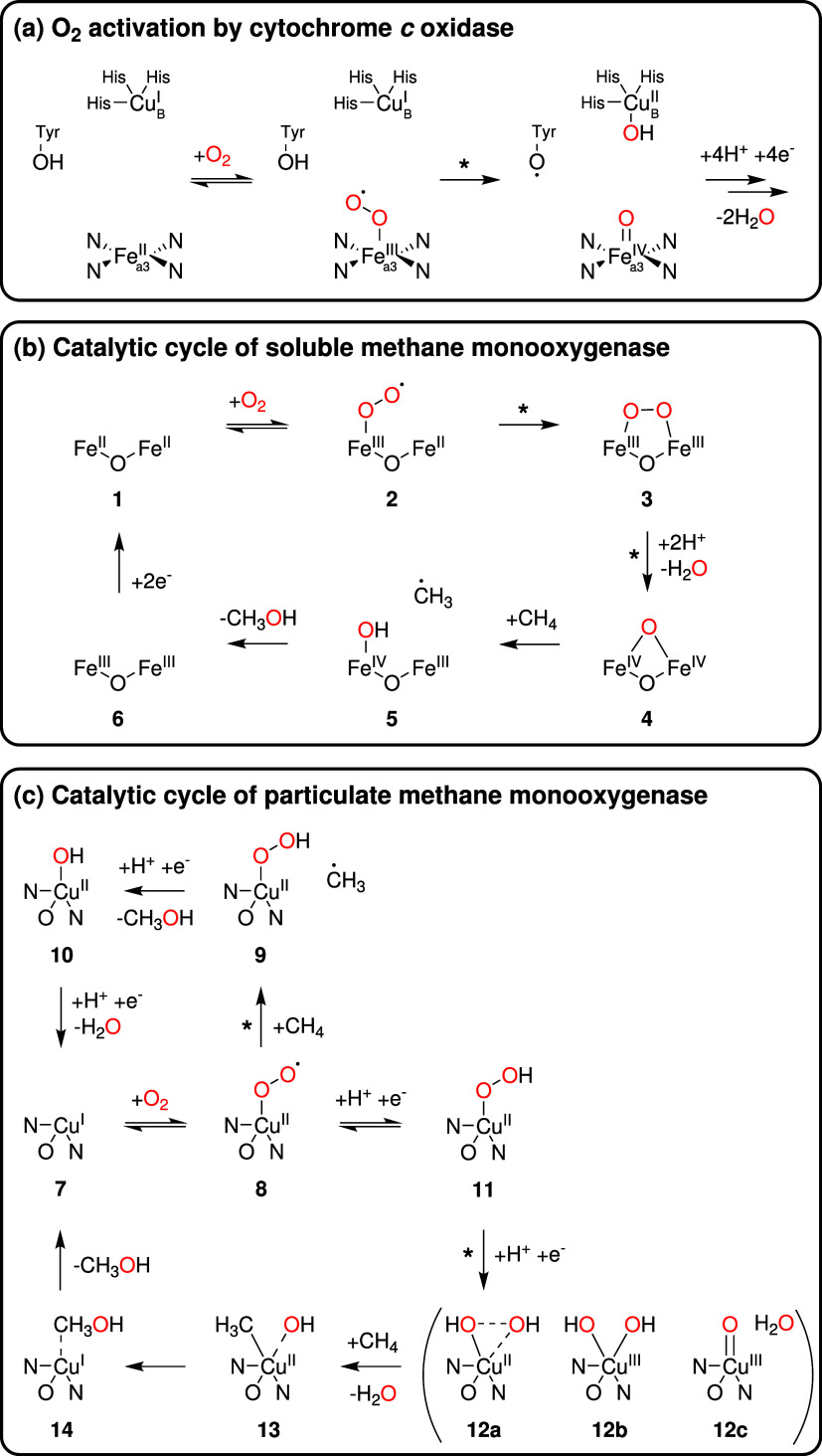
Key steps in the O_2_ activation mechanisms of (a) cytochrome *c* oxidase (adapted from ref 73, copyright 2015 American Chemical Society), (b) soluble methane monooxygenase (sMMO, adapted from ref 35, copyright 2024 American Chemical Society, and with permission under a Creative Commons CC-BY 4.0 license from ref 46, copyright 2001 ASBMB), and (c) particulate methane monooxygenase (pMMO, adapted from ref 35, copyright 2024 American Chemical Society and with permission under a Creative Commons CC-BY 4.0 license from ref 79, copyright 2024 P.E.M. Siegbahn). Potentially rate-limiting steps according to measured ^18^O kinetic isotope effects are marked with an asterisk (*).

The ^18^O-KIE determined for the diiron enzyme sMMO in this study with *M. silvestris* (1.022 ± 0.002) is larger than previously reported values for the isolated enzyme from *M. capsulatus* (1.015–1.017).^46^ While natural variability in isotope effects for a given enzyme is not uncommon,^76^ it is also possible that the isolated enzyme exhibits a different ^18^O-KIE outside its cellular environment, compared to in its *in vivo* state. In sMMO, it is proposed that O_2_ activation is initiated with a reversible O_2_ binding to one of the active-site iron atoms, followed by the formation of a diiron-bridged peroxo species (**3** in Figure 5b). One of the O atoms is subsequently released from the active site as H_2_O, while the other O atom may form a high-valent diiron-bridged oxo species (**4** in Figure 5b).^35^ The final steps in the catalytic cycle of sMMO involve hydrogen atom abstraction from CH_4_ by **4**, rebound of the methyl radical, release of methanol, and reduction of the active-site iron atoms (see Figure 5b).^35^ While Stahl et al.^46^ suggested the formation of a diiron-bridged peroxo species (**3**) to be the rate-limiting step based on their ^18^O-KIEs of 1.015–1.017, the value measured in this study (1.022 ± 0.002) is in better agreement with a rate-limiting formation of an iron-bound oxo species (**4**).^74^ A common rate-limiting iron-oxo species formation would also be compatible with the identical ^18^O-KIEs of sMMO and the terminal oxidase measured in this study. Further studies should focus to elucidate whether the difference in isotope effects of sMMO is due to experimental variables (*in vitro* vs *in vivo*) or the different bacterial species (*M. silvestris* vs *M. capsulatus*).

The ^18^O-KIEs determined for pMMO (1.023 ± 0.003) and AMO (1.019 ± 0.014) in this study align with the upper range of values previously reported for copper-dependent O_2_-consuming enzymes.^16^ Generally, O_2_ activation by copper-dependent enzymes with apparent ^18^O-KIEs between 1.017 and 1.022 is associated with a rate-limiting hydrogen atom abstraction, leading to the formation of a copper-hydroperoxo species.^75,77,78^ Given the similarities in substrate specificity, subunit compositions, and DNA sequences of pMMO and AMO, a common O_2_ activation mechanism is plausible. A complete catalytic cycle has not been resolved for either of the two enzymes, but experimental and computational evidence from studies with pMMO can be used, together with the measured ^18^O-KIEs, to suggest two possible O_2_ activation mechanism (see Figure 5C). While there is still debate regarding the exact location of the active site in pMMO, current evidence suggests a mononuclear copper center to be the active site (**7** in Figure 5c).^35^ In analogy to O_2_ activation mechanisms of other mononuclear copper enzymes with similar ^18^O-KIEs,^75^ the first step of the catalytic cycle likely involves reversible O_2_ binding and formation of a copper-superoxo intermediate (**8** in Figure 5c). This could be followed by a rate-limiting hydrogen atom abstraction by **8** directly at the substrate (CH_4_) and thus result in a Cu-hydroperoxo intermediate and a methyl radical (**9** in Figure 5c). The catalytic cycle can then be completed through a radical rebound step and the release of the products methanol and H_2_O. Computational studies, however, suggest that direct hydrogen atom abstraction from CH_4_, achieved enzymatically only by MMO and AMO, requires a more reactive intermediate, such as Cu­(II)-(hydr)­oxo or Cu­(III)-oxo species (**12a**–**12c** in Figure 5c).^79^ These species could form through a rate-limiting O–O bond cleavage step from a Cu-hydroperoxo intermediate (**11** in Figure 5c). While a Cu­(III)-oxo species has not yet been detected in enzymes,^35^ the analogous intermediate in iron-dependent enzymes (an Fe­(IV)-oxo species formed in a rate-limiting O–O bond cleavage step) is associated with a ^18^O-KIE of 1.022,^74^ similar to pMMO and AMO here. Similar to the mechanism mentioned above, this second possible catalytic cycle of pMMO (and AMO) is concluded with hydrogen atom abstraction from CH_4_, radical rebound, and release of the products methanol and H_2_O (see Figure 5c).^79^ The key difference between the two proposed mechanisms (**8** → **9** → **10** → **7** vs **8** → **11** → **12** → **13** → **14** → **7** in Figure 5) is the timing of hydrogen atom abstraction from CH_4_ relative to the first irreversible step of O_2_ activation. In the former pathway, hydrogen atom abstraction occurs during the first irreversible step, whereas in the latter, it occurs after the first irreversible step of the O_2_ activation. The nature of the rate-limiting step of pMMO could potentially be determined by comparing ^18^O-KIEs using protiated and deuterated substrates (CH_4_ or CD_4_). Deuteration of the substrate would alter the ^18^O-KIE onyl if hydrogen atom abstraction occurs during the first irreversible step of O_2_ activation.^80^ However, this analysis was beyond the scope of the current study. Nonetheless, the ^18^O-KIEs determined here for the first time for O_2_ activation by pMMO and AMO provide important evidence to better refine the catalytic cycle of these environmentally relevant enzymes.

## 4 Conclusions

The *in vivo* ^18^ϵ values for pMMO, sMMO, and AMO determined in this study ranged from −18 ± 12 ‰ to −24 ± 5 ‰. These values are not significantly different from the *in vivo* ^18^ϵ_resp_ values we report here for methanotrophs (−19.0 ± 0.7 ‰ to −22 ± 3 ‰) or from typical aquatic respiration ^18^ϵ values reported in previous studies (−18 to −24 ‰).^5,71,81,82^ This apparent similarity implies that O_2_ consumption by the widespread enzymes pMMO or AMO cannot account for the discrepancy observed between *in situ* and laboratory-derived ^18^ϵ values for O_2_ consumption in aquatic environments. Consequently, the ^18^ϵ values estimated for aerobic CH_4_ or NH_3_ oxidation indicate that these processes cannot be uniquely identified or quantified solely on the basis of O_2_ isotope analysis in the environment. Nevertheless, it would be interesting to conduct further experiments, particularly with other ammonia-oxidizers or mixed cultures, to test the robustness of the ^18^ϵ values determined in this study. We consider it unlikely that other biological (i.e., beyond respiration and CH_4_ or NH_3_ oxidation) or abiotic O_2_-consuming processes, such as H_2_S or Fe­(II) oxidation,^63,83,84^ which are characterized by low ^18^ϵ values, are pervasive enough in natural aquatic environments to significantly mask the isotopic imprint of heterotrophic O_2_ respiration at the ecosystem scale. And while photochemical reactions can have a significant impact on the isotopic fractionation of O_2_ consumption in surface waters,^85,86^ these processes unlikely mask the isotopic imprint of heterotrophic O_2_ respiration in lower parts of the water column, where *in situ* isotopic fractionation of O_2_ consumption is typically determined. Hence, the observed discrepancy between “community” *in situ* and laboratory ^18^ϵ values for O_2_ consumption is most plausibly explained by diffusion limitation at different spatial scales in natural environments or O_2_ consumption via biological ROS formation, as previously proposed.

## Data Availability

The data underlying this study are openly available at 10.5281/zenodo.17415999.
